# Autoimmunity as a novel mechanism underlying sarcopenia

**DOI:** 10.18632/aging.204703

**Published:** 2023-05-01

**Authors:** Tan Zhang

**Affiliations:** 1 Department of Internal Medicine, Section on Gerontology and Geriatric Medicine, Wake Forest School of Medicine, Winston-Salem, NC 27157, USA

**Keywords:** sarcopenia, skeletal muscle, autoimmunity, troponin T

Sarcopenia, the age-related decline in muscle mass and function, is a significant cause of disability and frailty in older adults and leads to poor quality of life and enormous health care costs. The pathogenesis of these changes is multifactorial and the related etiologies are not fully understood. A more complete understanding of the mechanisms driving these sarcopenic changes is required to identify both appropriate biomarkers and novel interventions to preserve and restore muscle mass and function in older adults and to improve healthspan and lifespan in older adults.

Growing evidence suggests that immunoglobulin G (IgG) mediated autoimmune process may be a critical, but under-recognized, contributor to sarcopenia. Autoimmunity increases with age [1] and two different meta-analyses strongly suggest a role of autoimmunity in sarcopenia and/or muscle weakness in older adults [2, 3]. Zhang et al. [4] further reported for the first time that IgG deposition/infiltration, usually a marker of increased membrane permeability and damage vulnerability, exists in skeletal muscle of older adults and aged mice in an IgG subclass and subcellular localization specific manner. Importantly, abundance of IgG1 but not IgG4 in skeletal muscle of older adults was negatively associated with physical performance (e.g. 400m walking speed). IgG subclass and subcellular localization specific deposition was also observed in aged C57BL/6 mouse, which strongly indicates that multiple autoantigens might be targeted in the aged skeletal muscle. The IgG subclass specificity is previously well-studied in Myasthenia Gravis (MG), an autoimmune skeletal muscle disease, where close to 90% of patients have IgG1 acetylcholine receptor autoantibodies and approximately 10% of MG patients have IgG4 MuSK autoantibodies. Identifying skeletal muscle autoantigens will be the key to understanding role of autoimmunity in skeletal muscle aging. Using transgenic mouse model with inducible skeletal muscle overexpression of cardiac troponin T (cTnT), Zhang et al. [4] revealed that inducible overexpression of cTnT in skeletal muscle could lead to appearance of anti-cTnT autoantibody in blood and lead to muscle degenerative or sarcopenic changes even at younger age stage. Importantly, anti-cTnT autoantibody was also found in blood of aged mice and older adults. This is consistent with a previously reported 9.9% prevalence of circulating anti-cTnT autoantibodies in 467 healthy human subjects aged from 18-72 [5]. Yet, the age depended prevalence of anti-cTnT autoantibodies and whether they are associated with sarcopenia still need to be determined.

Although detection of autoantibody is essential for diagnosis of any autoimmune diseases, a direct causal relationship has been demonstrated for several, but not all, autoimmune diseases [6]. As for anti-cTnT autoantibody, whether it has a direct causal effect on skeletal muscle damage in age mice, in transgenic skeletal muscle cTnT overexpression mouse, and in older adults is still unknown. Yet the fact that anti-cTnT autoantibody was not found to be among the recently identified 77 “common antibodies” discovered by a serum autoantibodyome meta-analysis [7] strongly supports a possible pathogenic role of anti-cTnT autoantibody. Technically, the causal effects of anti-cTnT or other skeletal muscle reactive IgG autoantibodies could be tested by passive transfer of isolated IgG autoantibodies in mouse *in vivo*, or treatment of isolated IgG on cultured skeletal muscle cell lines *in vitro*. Whether pathogenic or not, the newly discovered aging related IgG deposition/infiltration in skeletal muscle, at least as a marker of increased muscle fiber membrane permeability, could potentially be used as a novel biomarker to predict sarcopenia development and/or individual responses to clinical interventions. Similarly, anti-cTnT autoantibodies and any other skeletal muscle reactive autoantibodies to be discovered in blood of older adults may also serve as novel biomarkers for sarcopenia diagnosis and development prediction. Autoimmunomic signatures of aging therefore could be of great potential to identify novel sarcopenia related circulating biomarkers in older adults, as was found in age-related neurodegenerative diseases [8].

Based on IgG infiltration in skeletal muscle and/or skeletal muscle reactive autoantibodies in blood, a possible new subtype of sarcopenia could be established, which could be originated from age-dependent plasma membrane senescence with or without subsequent autoimmune responses. Genetic and cellular/molecular mechanism underlying this age-related membrane integrity impairment and autoimmunity in skeletal muscle will need to be explored for better developing new preventive and therapeutic strategies on sarcopenia prevention and intervention. Therefore, epidemiology study will be the next step to identify skeletal muscle reactive autoantibodies and determine their prevalence and association with sarcopenia in older adults. Together with basic research studies in aging mouse models and transgenic mouse models, a more in-depth understanding of mechanisms, risk factors, and clinical implications of autoimmunity in sarcopenia pathogenesis and development will guarantee rich practical rewards.
